# Low knowledge and awareness of monoclonal gammopathy of undetermined significance (MGUS) among general practitioners

**DOI:** 10.1186/s12875-019-0944-5

**Published:** 2019-05-14

**Authors:** Charlene M. McShane, Blain Murphy, Olinda Santin, Lesley A. Anderson

**Affiliations:** 1 Cancer Epidemiology Research Group, Centre for Public Health, Queen’s University Belfast, Grosvenor Road, Belfast, BT12 6BJ Northern Ireland; 2 School of Nursing and Midwifery, Queen’s University Belfast, Belfast, Northern Ireland

**Keywords:** MGUS, Healthcare professionals, Haematology, Myeloma, Communication aids, Family doctors/primary care

## Abstract

**Introduction:**

While multiple myeloma (MM) is a rare diagnosis within primary care, its precursor MGUS (monoclonal gammopathy of undetermined significance) is more common, particularly among older populations. Upon first detection, the majority of MGUS patients will be under the care of their General Practitioner (GP)/Family Doctor who is also often the first healthcare professional that patients report symptoms of progression to. However, our previous work with MGUS patients and haematology healthcare professionals has suggested that knowledge and awareness of MGUS is low among GPs.

**Methods:**

An online survey was undertaken to investigate knowledge and awareness of MGUS and services needed by GPs/GP trainees to support these patients. The survey was promoted at a large European primary care conference and via social media. Descriptive statistics were utilised to compare participant responses.

**Results:**

In total 58 GPs (*n* = 35 GPs and *n* = 23 GP trainees) from 24 countries responded. Overall, self-reported familiarity with the term MGUS was low (mean score: 2.21/5, standard deviation (SD): 1.09), but higher among GPs who reported having at least one MGUS patient (mean score: 2.83/5, SD 0.99). The majority (88.2%) of GPs/GP trainees stated they would feel uncomfortable discussing MGUS with patients. The increased risk of haematological malignancies was identified by 62.1% of GPs/GP trainees with MM, lymphoma and myelodysplastic syndromes the most commonly reported cancers associated with MGUS. The majority (81.6%) of GPs/GP trainees were supportive of patient follow-up via telephone clinics (phlebotomy performed in GP practice with patient management maintained by haematology) but only 27.1% stated they would be happy to solely manage all low/low-intermediate risk MGUS patients. A laboratory report alerting to the possibility of MGUS or a haematological malignancy was reported as the most useful service which could be implemented to help GPs manage MGUS patients. The need for MGUS focused information and education resources for GPs was also highlighted.

**Conclusions:**

The findings of this study highlight a lack of knowledge and awareness of MGUS among GPs/ GP trainees. The majority of GPs/GP trainees are happy to support haematology in managing these patients but require assistance and support in providing these services.

## Background

Multiple myeloma (MM), an incurable B-cell malignancy [1] is the third most common haematological malignancy diagnosed worldwide [2]. MM is proceeded by monoclonal gammopathy of undetermined significance (MGUS) [3, 4], which is estimated to be present in 3.2% of the population aged 50 years and older [5]. Owing to its asymptomatic nature, MGUS is markedly under diagnosed and is often detected incidentally upon routine blood testing [6, 7]. Clinically, MGUS is defined, by the International Myeloma Working Group, as < 30 g/l of serum monoclonal (M) protein, < 10% plasma cell infiltration in the bone marrow and absence of end organ damage (CRAB criteria – hypercalcaemia, renal insufficiency, anaemia and bone lesions) [8]. The annual rate of progression to MM and related haematological malignancies is between 0.5–1%, and remains elevated beyond 25 years of observation [9, 10]. Follow-up guidelines for MGUS vary internationally, however, most advocate for one annual follow-up visit with relevant myeloma-related investigations [6, 11–13]. In general, it is recommended that these follow-up visits continue indefinitely or until life expectancy becomes limited [6, 11–13].

The majority of patients detected with an M-protein will initially be under the care of their general practitioner (GP)/Primary Care Physician or a clinician outside haematology [11]. Previous research by the study team investigating the psychosocial impact of MGUS among patients has highlighted low awareness and knowledge of MGUS among healthcare professionals outside haematology and in particular, among their GP (*Unpublished findings*). In response to these findings, the study team undertook a short survey of haematology doctors and nurses attending the Haematology Association of Ireland meeting in October 2016 [14]. Similar findings to the patient study were reported, with haematology healthcare professionals highlighting confusion among patients and GPs alike [14]. Of note, many haematology healthcare professionals reported a combined approach to follow-up involving primary and secondary care is now needed to deal with the increasing number of low/low-intermediate risk MGUS patients being diagnosed [14]. Respondents also recognised that GPs should be supported in this role and provided with guidelines to avoid over-diagnosing and over-referring patients to haematology [14]. Within the current study, we explored GP knowledge and awareness of MGUS and their perceived support needs to manage MGUS patients within primary care.

## Methods

GPs/trainees attending the 22nd WONCA (World Organisation of National Colleges, Academics and Academic Associations of General Practitioners/Family Physicians) Europe conference in Prague, Czech Republic (http://www.woncaeurope2017.eu/) were invited to participate in an online survey, detailed below. The WONCA Europe Conference is an annual Europe-wide GP/family doctor conference attended by GPs and GP trainees from across the world.

### Survey

In the absence of a validated questionnaire, a short online survey was developed informed by the study team’s previous studies with patients and haematology healthcare professionals. The survey was hosted on SurveyMonkey®. The survey consisted of 35 questions developed by the study team to capture information relating to awareness and knowledge of MGUS and potential support needs of GPs/GPs in training. The first seven questions were designed to capture respondent demographics and the remaining questions related specifically to MGUS. Familiarity with MGUS was assessed using a likert scale of 1–5 with 1 equivalent to ‘no knowledge/never heard of it’ and 5 being ‘very familiar’. Multiple choice questions and skip logic were used to reduce the time taken to complete the survey (median time for completion: 9 min). All respondents were required to answer the multiple choice/tick box questions but could leave open-ended questions blank. All responses to the survey were anonymous. To increase awareness of the survey, study posters, leaflets and promotion slides were used to advertise the study during the conference. The study team set up a twitter account (@QUB_GPsurvey) and a promotion link was included on the WONCA Europe website. Respondents were provided with the option of being included in a draw for a Samsung Galaxy® tablet.

## Data analysis

The returned survey responses were transferred directly from SurveyMonkey® into Microsoft Excel to facilitate data cleaning. The data was analysed to investigate GP awareness and knowledge of MGUS and the support services needed to manage this group of patients. We excluded one respondent from the analysis who stated in the survey that they were not a GP/GP trainee. Descriptive statistics and Chi-squared and Fisher’s Exact tests were used to compare participant responses based on variables of interest including GP status (registered GP vs trainee), world region and number of MGUS patients within practice. To assess awareness of MGUS, mean scores and standard deviation were calculated based on a self-reported scale ranging from 1 to 5 with 1 being ‘unfamiliar’ and 5 being ‘very familiar’ with MGUS. Missing data was coded as a specific category and excluded from the denominator when calculating responses to questions asked. Responses to open-ended questions were reviewed and analysed using content analysis [15]. All tests were two tailed and a *p*-value < 0.05 was considered statistically significant. All analyses were performed using STATA (version 14, StataCorp, TX, USA).

### Ethical approval

This study received ethical approval from the School of Medicine, Dentistry and Biomedical Sciences Research Ethics Committee, Queen’s University Belfast (Ref 17.22).

## Results

### Overview of respondents

In total, 58 GPs/GP trainees from 24 countries responded to the online survey, Table 1. Of the 58 respondents, the majority were male (55.2%), practicing GP’s/Family Practitioners (60.3%) and had completed their medical degree within the last 5 years (43.1%). As expected, the majority (*n* = 47; 81%) of respondents came from European countries (Portugal *n* = 9, United Kingdom *n* = 6, Netherlands *n* = 6, Spain *n* = 6, Ireland *n* = 4, Greece *n* = 3, Croatia *n* = 3, Lithuania *n* = 2, Luxembourg n = 2, Serbia *n* = 2, Romania *n* = 1, Finland *n* = 1, Sweden *n* = 1, Switzerland *n* = 1). Six (10.3%) respondents came from Asia (Hong Kong *n* = 1, Indonesia *n* = 1, Israel *n* = 1, Lebanon *n* = 1, Saudi Arabia *n* = 1 and Turkey *n* = 1) while three (5.2%) came from The Americas (*n* = 1 USA, *n* = 1 Brazil and *n* = 1 Mexico) and one from Africa (Tunisia). The majority of respondents worked in metropolitan/urban areas (72.4%) and served more than 1000 patients (89.7%). Just over half of the respondents (*n* = 30; 51.7%) reported knowing that they had at least one MGUS patient enrolled within their GP practice.

**Table 1:** GP/GP trainee respondent demographics

	Total (*n* = 58)	Registered GP (*n* = 35)	GP trainee (*n* = 23)	*p*-value*
Gender				
Male	32 (55.2)	22 (62.9)	10 (43.5)	0.15
Female	26 (44.8)	13 (37.1)	13 (56.5)	
Continent				
Europe	47 (81)	27 (77.1)	20 (87)	0.54
The Americas	3 (5.2)	1 (2.9)	2 (8.7)	
Asian	6 (10.3)	5 (14.3)	1 (4.4)	
Africa	1 (1.7)	1 (2.9)	0	
Missing	1 (1.7)	1 (2.9)	0	
Years working as a GP or trainee since completing medical degree				
0–5 years	25 (43.1)	5 (14.3)	20 (87)	≤0.001
6–10 years	13 (22.4)	11 (31.4)	2 (8.7)	
11–20 years	13 (22.4)	13 (37.1)	0	
20+ years	7 (12.1)	6 (17.1)	1 (4.4)	
GP Practice				
Metropolitan/urban	42 (72.4)	25 (71.4)	17 (73.9)	0.52
Rural	15 (25.9)	10 (28.6)	5 (21.7)	
Prefer not to say/Not applicable	1 (1.7)	0	1 (4.4)	
Number of patients within GP/Family practice				
0–500 patients	3 (5.2)	1 (2.9)	2 (8.7)	0.83
501–1000 patients	3 (5.2)	2 (5.7)	1 (4.4)	
1001–2000 patients	25 (43.1)	16 (45.7)	9 (39.1)	
2001+ patients	27 (46.6)	16 (45.7)	11 (47.8)	
Number of MGUS patients that have/are currently within GP/Family practice				
None	8 (13.8)	5 (14.3)	3 (13)	0.19
1–10 patients	24 (41.4)	18 (51.4)	6 (26.1)	
11–50 patients	5 (8.6)	2 (5.7)	3 (13)	
51–100 patients	1 (1.7)	1 (2.9)	0	
Don’t know/Prefer not to say/Not applicable	20 (34.5)	9 (25.7)	11 (47.8)	

*Fisher’s exact test used to estimate *p*-value where cell count < 5

### Knowledge and awareness of MGUS

When asked to rank familiarity with the term MGUS on a scale of 1–5 (with 1 being unfamiliar and 5 being very familiar), the respondents reported a mean score of 2.21, standard deviation ±1.09. Registered GPs reported slightly greater familiarity than GP trainees (mean 2.23 vs 2.17) but this difference was not statistically significant. GPs/GP trainees who reported having at least one MGUS patient within their practice reported greater familiarity with MGUS compared to those that did not report any patients or selected ‘Prefer not to say’ (2.83 ± 0.99 vs 1.54 ± 0.74; *p*-value≤0.001). The highest mean familiarity score was observed for those GP’s/trainees who reported having 11–50 MGUS patients within their practices (3.4 ± 1.14) although this finding was based on *n* = 5 participants. While there were no significant differences between world regions, respondents from Europe reported being more familiar with MGUS [mean score (2.26 ± 1.11)] compared to respondents from Asia (2 ± 1.26), the Americas (1.67 ± 0.58), and Africa (*n = 1 participant; data not reported to maintain confidentiality)*.

**Fig. 1:**
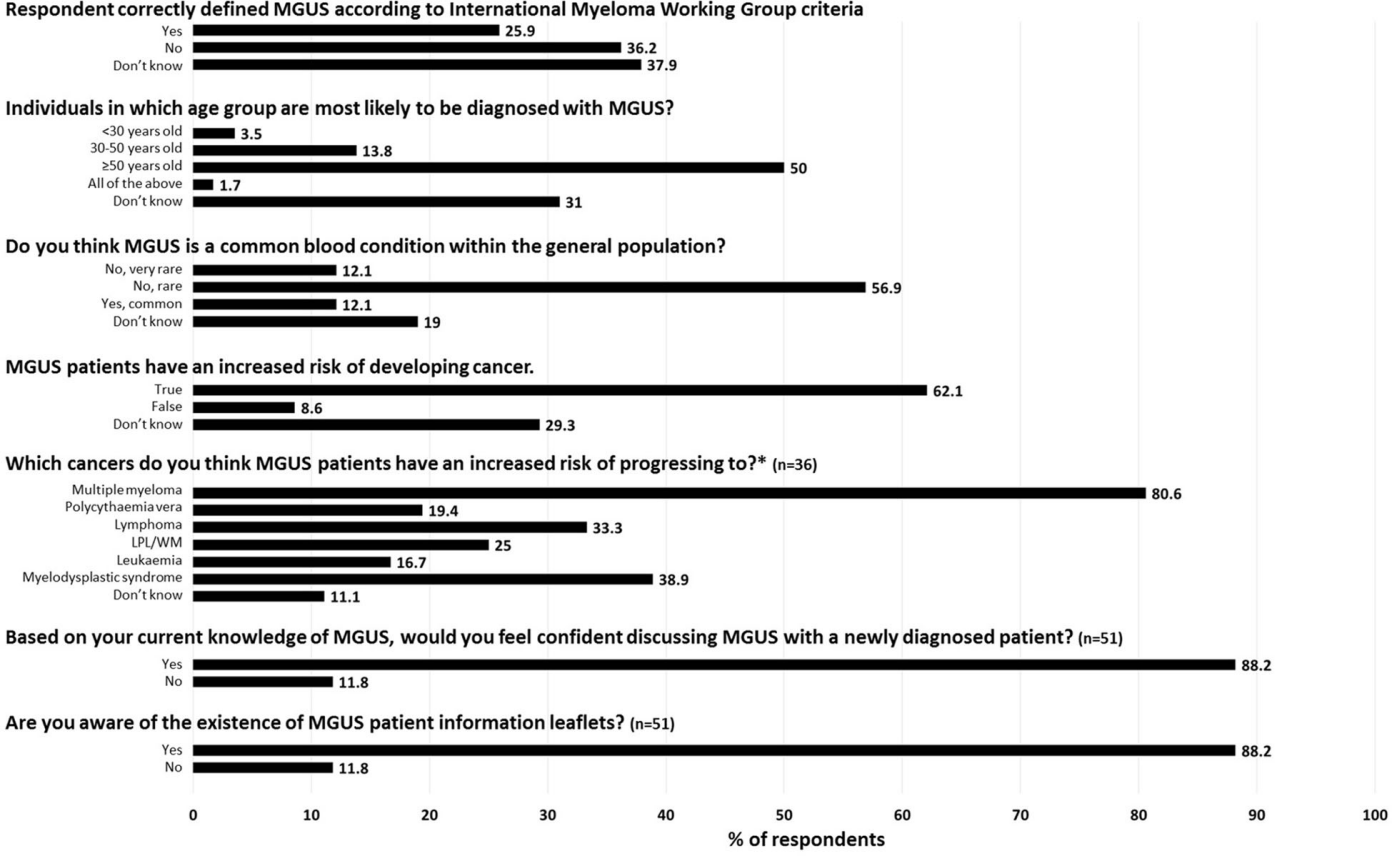
Awareness and knowledge of MGUS among GP/GP trainee respondents. Percentages are based on responses from 58 GP/GP trainees unless otherwise stated by the numbers in brackets. *respondents were allowed to select more than one option and so percentages may not add up to 100%. Abbreviations: LPL/WM: lymphoplasmacytic lymphoma/Waldenström’s macroglobulinemia

While just over half (53.5%) of the respondents recognised MGUS as being a pre-malignant blood disorder associated with production of monoclonal protein, only 25.9% (*n* = 15) of respondents correctly identified MGUS using the International Myeloma Working Group’s definition (serum monoclonal protein < 30 g/L, clonal plasma cells < 10% and absence of end organ damage associated with the underlying plasma cell disorder, Fig. 1). GPs/GP trainees with at least one MGUS patient were more likely to answer correctly compared to respondents who reported having no MGUS patients (or prefer not to say) [*n* = 12 (40%) vs *n* = 3 (10.7%); *p*-value = 0.006]. GPs/GP trainees without experience of MGUS patients (or who answered ‘prefer not to say) were more likely to report their answer as ‘Don’t know’ compared to respondents with MGUS patients [*n* = 16 (57.1%) vs *n* = 6 (20%)].

The majority of participants reported MGUS to be a rare blood disorder (*n* = 33; 56.9%) occurring in those aged 50 years and older (*n* = 29; 50%), Fig. 1. Of the 58 participants, 62.1% (*n* = 36) correctly identified that MGUS is associated with an increased risk of malignancy. GPs/GP trainees with at least one MGUS patient were more likely to answer correctly (*n* = 23; 76.7%) compared to respondents who reported having no experience with MGUS patients or prefer not to say (*n* = 13; 46.4%; *p*-value = 0.02). The respondents most commonly associated MGUS with progression to MM (*n* = 29/36 respondents; 80.6%), myelodysplastic syndromes (*n* = 14; 38.9%) and lymphoma (*n* = 12; 33.3%). Of the 40 GPs/trainees who responded to the question on signs and symptoms, 57.5% (*n* = 23) reported being aware of signs/symptoms associated with progression. Of the 19 respondents who provided additional detail to this question, the most frequent signs/symptoms associated with progression were reported to be bone pain/lesions/osteoporosis (*n* = 10; 52.6%), renal disease/abnormalities (*n* = 7; 36.8%), infections (*n* = 5; 26.3%), fever (*n* = 4; 21.1%), anaemia (*n* = 3; 15.8%), bleeding (n = 3; 15.8%), weight loss (*n* = 3; 15.8%), fatigue (*n* = 2; 10.5%), weakness (*n* = 2; 10.5%), increase in M protein/worsening of blood tests (*n* = 2; 10.5%), night sweats (*n* = 1; 5.3%), and hypercalcaemia (*n* = 1; 5.3%).

### MGUS follow-up

Of the 51 participants who completed questions on MGUS follow-up, 88.2% (*n* = 45) stated that based on their current level of knowledge they would not feel comfortable discussing MGUS with a newly diagnosed patient, Fig. 1. This finding did not differ by the number of MGUS patients within the GP practice, however GP trainees were more likely to report feeling uncomfortable (*n* = 19; 100%) compared to practising GP’s (*n* = 26; 81.3%; *p*-value = 0.04). Of the 15 respondents who provided additional information, 11 (73.3%) stated that they were uncomfortable due to their limited knowledge/experience of MGUS and would need to update their knowledge before advising patients. While the majority of GPs/GP trainees reported providing information leaflets to some or all of their patients in general (i.e. any medical diagnosis), only 11.8% (*n* = 6) were aware of the existence of MGUS information leaflets, Fig. 1.

**Fig. 2:**
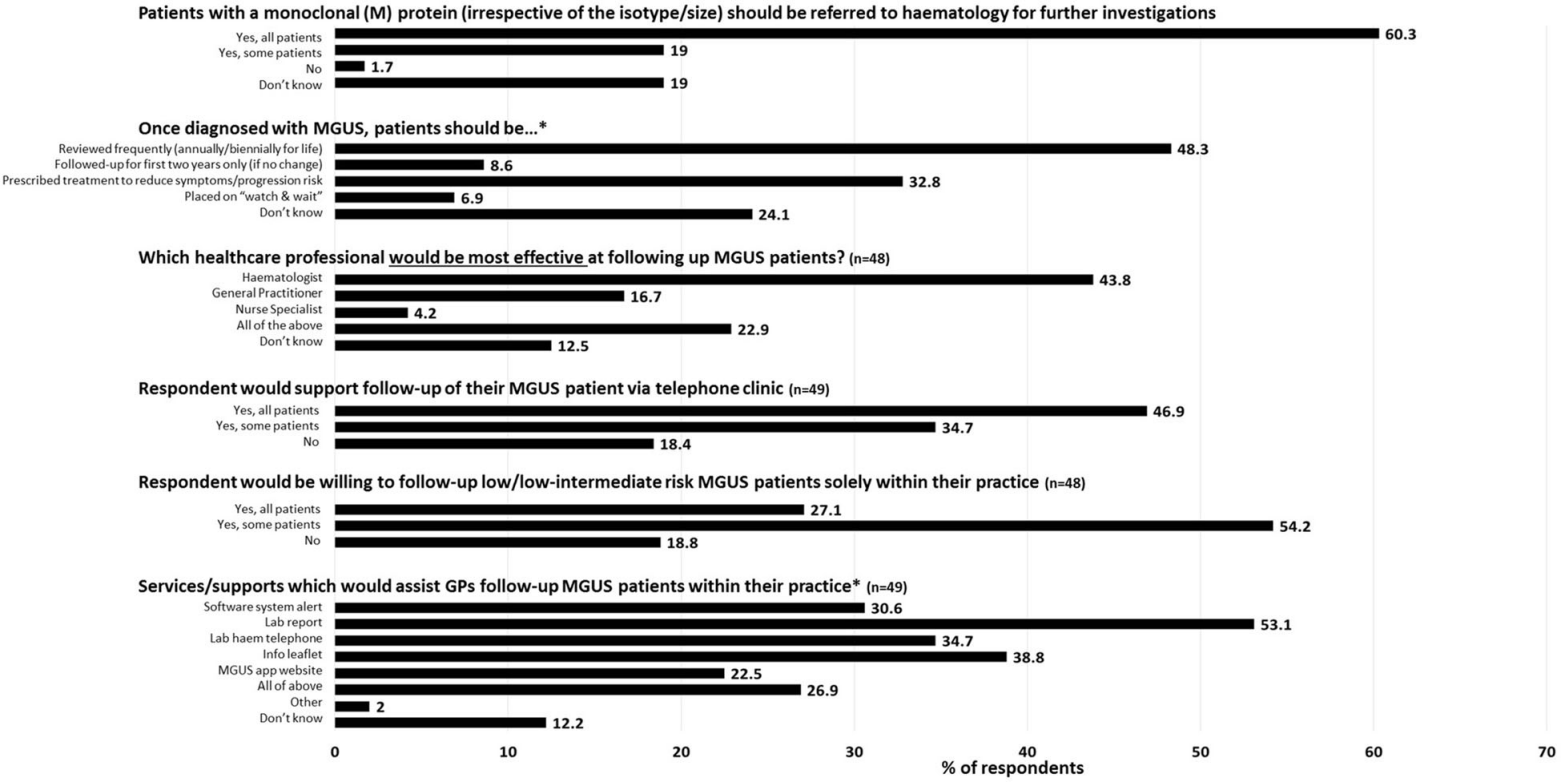
GP/GP trainees views towards MGUS follow-up, percentages are based on responses from 58 GP/GP trainees unless otherwise stated by the numbers in brackets. *respondents were allowed to select more than one option and so percentages may not add up to 100

Of the 58 participants, 46 (79.3%) stated that they would either refer all or some patients to haematology if a paraprotein (irrespective of size/isotype) was identified, Fig. 2. Of the 17 respondents who provided additional information, reasons for referral included for diagnostic confirmation and for ruling out malignancy. Some respondents (*n* = 5) stated they would refer patients due to their limited knowledge of MGUS, with one GP stating “*Interpreting monoclonal antibodies is more specialised and would be outside a GPs competence to interpret these.*” While the majority (*n* = 21/48 respondents; 43.8%) deemed haematologists to be the most effective healthcare professionals at following-up MGUS patients, 23% (*n* = 11) reported a combination approach consisting of haematologists, GPs and nurses as the best strategy. Of the 49 respondents who completed questions regarding follow-up, 40 (81.6%) were happy for (all or some) MGUS patients to be followed-up via a telephone clinic with a haematology nurse/haematologist while just 27.1% (*n* = 13/48 respondents) stating that they would be happy to follow-up all low/low-intermediate risk MGUS patients within their own GP practice, Fig. 2. Respondents highlighted the need for clear guidelines and guidance from haematology in order to facilitate follow-up. Additional issues highlighted included GP workload and patient safety, for example missing follow up or progression to cancer.

When asked, “What services/information should be provided to GPs/Family Physicians to help them manage MGUS patients?”, 53.1% (*n* = 26/49 respondents) of GPs/GP trainees considered laboratory reports alerting them to the possibility of MGUS or a haematological malignancy as being the most useful service which could be implemented, Fig. 2. Other additional services which ranked highly included, information leaflets (38.8%; *n* = 19), and/or receiving a phone call (34.7%; *n* = 17) from the haematology team at the point of patient diagnosis, and/or an alert by the clinical software system (30.6%; *n* = 15). Of the 26 respondents who provided additional information, *n* = 15 (57.7%) highlighted the need for information and education resources in the form of information leaflets, journal articles, seminars, webinars, courses and a haematology run website. Respondents were particularly interested in learning more about diagnosis, risk assessment, follow-up, management and signs/symptoms considered red flags. An additional *n* = 6 respondents (23.1%) highlighted their preference for having direct access to a haematology specialist or helpdesk when necessary.

## Discussion

MM is a rare diagnosis within primary care; the average GP in the United Kingdom (UK) seeing one new MM patient every 8–10 years [16]. It is perhaps not surprising that our survey revealed a low level of knowledge and awareness of MGUS, the MM precursor among GPs/trainees. These findings are also in agreement with our previous studies with MGUS patients and haematology healthcare professionals which reported low awareness of MGUS outside of haematology [14]. Despite this, there is increasing government and public demand for primary care to expand its role in cancer prevention, early detection and control, and management within the community [16]. The challenge therefore remains to increase GP awareness and knowledge of MM and its precursor MGUS.

Of the 24 most common cancer sites, MM has previously been associated with the highest percentage of patients visiting their GP three or more times prior to diagnosis [17]. Within the UK, 37% of MM cases are still diagnosed within emergency care and have been reported to experience poorer outcomes compared to those patients recognised and referred to secondary care by their GPs (one-year survival for GP referral vs emergency presentation: 81% vs 51% respectively) [18]. Classified as a ‘hard to suspect’ cancer [19], MM patients typically present with a myriad of symptoms of a non-specific nature, including, bone pain and extreme fatigue [20]. While just under 60% of GP’s/trainees in this study reported knowing the sign/symptoms associated with MGUS progression, a number of GP’s/trainees respondents stated that this area was outside of their expertise and that they would feel uncomfortable discussing the MGUS diagnosis with their patient. The pathway to cancer diagnosis, relies on a relationship of trust between the patient and the doctor. The patient must first be aware of the signs/symptoms to look out for, present to their doctor and their doctor must recognise the possibility of cancer. This relationship is more complex within the UK and similar healthcare systems, where the GP is the gate-keeper to secondary care and specialist treatment. While GPs are not expected to be experts in the field, limited knowledge can lead to delayed identification and negatively impact their patients [17]. Howell and colleagues (2015) previously reported blood cancer patients who did not believe their symptom(s) to be serious were more likely to delay presenting to their doctor [21].

While a ‘watch and wait’ system is currently recommended for MGUS and smouldering MM patients, recent evidence suggests that initiating treatment early in the MM pathway may have beneficial effects [22]. Furthermore, in a recent Swedish study, MM patients with a prior knowledge of MGUS were reported to have better overall survival compared to patients without a prior MGUS diagnosis [23]. While not possible to establish a causal link between follow-up and survival benefits from this single study, the authors suggest that the findings highlight the importance of clinical follow-up for all MGUS patients, irrespective of risk stratification. However, as a reasonably common phenomenon detected within older populations [5], the number of individuals diagnosed with MGUS is placing an increasing burden on secondary care haematology services. Our recent survey of haematologists in the UK and Irish healthcare systems supported a combined primary and secondary care effort to manage MGUS patient follow-up. The findings from the present study suggests that while this approach would have support from GPs/GP trainees, just over a quarter of respondents were happy to follow-up all low/low-intermediate risk MGUS patients within their GP practice. Within several areas in the UK, outreach haematology monitoring services have been set up for conditions including MGUS and have been reported to improve patient satisfaction and reduce the burden on secondary care [24]. Compliance by general practice for this service has been reported to be very high [24]. This survey highlights an opportunity for haematology specialists to assist GPs’ in managing MGUS patients through the provision of clear follow-up guidelines. In addition, laboratory reports (highlighting MGUS or a possible blood malignancy), information leaflets and/or telephone call by haematology team were ranked as the most effective means of communicating with primary care regarding MGUS. Future research including the development of working groups involving primary care and haematology specialists could assist in identifying areas of misunderstanding and in developing relevant resources.

This study is the first to assess knowledge and awareness of MGUS among GPs/primary care physicians. While the generalisability of our findings may be limited by the sample size, we report findings from general practitioners/trainees working in 24 countries and across 4 continents’. Furthermore, it is well recognised that GPs are a difficult group to research owing to their work time constraints. The online nature of the survey and restriction to English speakers may have further impacted the generalisability of the findings however, the survey was promoted at a European general practitioner conference and via social media. Similar responses were reported by all respondents suggesting that the findings are representative and could be applied to other regions and healthcare services.

## Conclusions

In conclusion, the findings of this study highlight a lack of knowledge and awareness of MGUS among general practitioners/trainees. These findings are important as the number of MGUS patients detected continues to increase and follow-up potentially transitions towards primary care. Our survey suggests the majority of GPs/trainees are happy with this transition but require assistance and support from haematology in providing these services.

## Abbreviations

GP: General Practitioner
MGUS: Monoclonal gammopathy of undetermined signficiance
MM: Multiple myeloma
SD: Standard deviation
UK: United Kingdom
WONCA: World Organisation of National Colleges, Academics and Academic Associations of General Practitioners/Family Physicians
